# Quality of life and nutritional status in peritoneal dialysis patients: a cross-sectional study from Palestine

**DOI:** 10.1186/s12882-023-03422-9

**Published:** 2024-01-12

**Authors:** Iyad Ali, Dania Haddad, Mostafa A. Soliman, Ahmed Al-Sabi, Kamel Jebreen, Dana Abuzahra, Bakrieh Shrara, Diana Ghanayem, Nihal Natour, Mohanad Hassan, M. Yasser Alsedfy, Duha Shellah, Inad Nawajah

**Affiliations:** 1 Department of Medical Sciences, Faculty of Medicine and Health Sciences, An-Najah National University, Nablus, Palestine; 2 Department of Genetics and Bioinformatics, Dasman Diabetes Institute, Dasman, 15462 Kuwait; 3 Faculty of Medicine, Cairo University, Cairo, Egypt; 4 College of Engineering and Technology, American University of the Middle East, Egaila, 54200 Kuwait; 5 Unité de Recherche Clinique Saint-Louis Fernand-Widal Lariboisière, APHP, Paris, France; 6 Department of Mathematics, An-Najah National University, Nablus, Palestine; 7 Department of Mathematics, Palestine Technical University Kadoorie, Hebron, Palestine; 8 Alexandria University Cancer Research Cluster, 21561 Alexandria, Egypt; 9 Department of Mathematics, College of Science and Technology, Hebron University, Hebron, Palestine

**Keywords:** End-stage renal Disease, Malnutrition, Nutrition-inflammation scale, Peritoneal dialysis, Quality of life, Palestine

## Abstract

**Background:**

End-stage renal disease (ESRD) is a growing cause of morbidity worldwide. Protein malnutrition is common among patients with ESRD. Peritoneal dialysis (PD) offers greater lifestyle flexibility and independence compared to the widely used treatments for ESRD. This study aimed to assess the nutritional status and the quality of life (QOL) of Palestinian patients undergoing PD, as well as the variables affecting these two outcomes.

**Methods:**

A cross-sectional study was conducted on patients receiving PD at An-Najah National University Hospital, Palestine. The malnutrition-inflammation scale (MIS) was used to measure malnutrition, and the QOL score was evaluated using the Dutch WHOQOL-OLD module. Univariate and multivariate linear regressions were performed to check the association between the QOL and MIS scores.

**Results:**

The study included 74 patients who were undergoing PD, with an average age of 50.5 ± 16.38. The majority of the patients were females. The study found a significant correlation between malnutrition and lower quality of life (QOL) scores, as measured by the WHOQOL-OLD questionnaire (*p* < 0.001). Furthermore, younger patients and those who had an occupation were more likely to report a good QOL (*p* = 0.01). Conversely, patients with pitting edema and diabetes were at higher risk of reporting a lower QOL (*p* < 0.001).

**Conclusions:**

Given the elevated risk of malnutrition and diminished QOL among elderly patients, those with pitting edema, and individuals with diabetes, it is imperative to conduct thorough assessments for these groups. We strongly recommend that general practitioners, dietitians, and specialists collaborate to develop tailored programs and interventions to provide these patients with the focused care and attention they require.

## Background

Chronic kidney disease (CKD) is a progressive condition characterized by the gradual deterioration of kidney function over time. If left untreated, CKD can advance to end-stage renal disease (ESRD). CKD is typically diagnosed when there is evidence of kidney damage or reduced kidney function, as indicated by a glomerular filtration rate (GFR) below 60 mL/min/1.73 m², persisting for at least three months, regardless of its underlying cause. On the other hand, ESRD represents the most advanced stage of chronic kidney disease, resulting from a profound loss of kidney function, with a GFR falling to less than 15 mL/min/1.73 m² and lasting for at least three months. At this point, referred to as Grade 5 [1], the primary treatment option becomes dialysis.

ESRD represents a global health challenge, affecting thousands of patients and placing a significant burden on healthcare systems. This condition leads to a buildup of waste products in the blood, electrolyte imbalances, and fluid overload, all of which have profound consequences for patients. There are several treatment options available for ESRD, including hemodialysis (HD), peritoneal dialysis (PD), and kidney transplantation. These treatment approaches are primarily geared toward slowing the progression of the disease and addressing its associated complications. While kidney transplantation, though effective, is a complex and costly procedure that relies on the availability of suitable donors, dialysis has emerged as the primary treatment modality for ESRD patients [2].

CKD has numerous causes, including type 2 diabetes mellitus (T2DM), hypertension, chronic use of anti-inflammatory medications, chronic glomerulonephritis, and autoimmune diseases [3]. The prevalence of CKD cases is on the rise, primarily due to the high incidence of non-communicable diseases, such as T2DM and hypertension, which can lead to kidney failure. The World Health Organization defines quality of life (QOL) as an individual’s perception of their position in life within the context of their culture and value systems, as well as in relation to their goals, expectations, standards, and concerns [4]. On the other hand, malnutrition is characterized by deficiencies, excesses, or imbalances in an individual’s energy and nutrient intake, which can impact their overall health [5]. In PD, the catheter insertion procedure involves placing the patient in a supine position, and whether general or local anesthesia is administered depends on the patient’s specific medical condition [6].

Continuous ambulatory peritoneal dialysis (CAPD) is a dialysis method that does not require the use of a machine. Patients typically need to perform at least three sessions daily during their waking hours. CAPD offers the advantage of enabling patients to manage their dialysis regimen from the comfort of their homes or workplaces. In contrast, automated peritoneal dialysis (APD), also known as continuous cycling peritoneal dialysis, relies on a machine called a cycler to carry out each dialysis session. Patients can opt for a single extended session using the cycler while they sleep or multiple shorter sessions throughout the day [7]. Adjusting to dialysis schedules can significantly impact patients’ daily lives and overall well-being, affecting their social, physical, and psychological aspects [8].

A patient’s QOL is influenced by various factors, including their functioning, happiness, and perceptions of health across physical, psychological, and social domains [9]. In chronic diseases, especially CKD, QOL, morbidity, and mortality are closely intertwined [10, 11].

Studies have shown that CKD patients tend to have significantly lower QOL compared to healthy individuals, with this difference being more pronounced in the pre-dialysis stage, especially among older patients [12, 13]. Reduced functional status and QOL often coincide with declining GFR and an increase in uremic symptoms such as anorexia, weakness, fatigue, and muscle cramps [14]. Research has also indicated that nutritional status plays a role in dialysis patients’ QOL, although the existing body of evidence on dietary management’s impact remains limited.

The approach to QOL in CKD patients, particularly those with end-stage renal disease, has shifted from merely ensuring survival to fostering a sense of well-being [15]. The constraints imposed by CKD treatment frequently lead to a decline in QOL, which can be exacerbated by comorbidities and other health conditions. As the clinical condition and QOL of these individuals are closely linked to their overall health and survival, interventions are necessary to enhance their well-being. The connection between declining QOL and potentially manageable factors such as diabetes [16], aging [17], suboptimal dialysis, inflammation, and nutrition remains a topic of ongoing research. Initiation of dialysis has demonstrated varying effects on the QOL of end-stage renal disease (ESRD) patients, and studies have reported mixed results in the relationship between QOL and nutritional parameters due to limitations in patient sample sizes, non-ESRD-specific assessments, observation durations, and other variables [18, 19].

Numerous disease-specific health-related quality of life (HRQOL) questionnaires have been developed and validated for the dialysis population, including the Choices Health Experiences Questionnaire (CHEQ), World Health Organization Quality of Life Survey (WHOQOL), Kidney Disease Quality of Life (kDQOL), and Short Form (SF)-36 health survey.

Numerous studies have extensively examined the QOL in patients with CKD who are undergoing renal replacement therapy, with a particular focus on transplant recipients and HD patients. In contrast, previous research on PD patients is notably limited. Therefore, the primary goal of this study is to evaluate the impact of PD on both patients’ nutritional status and overall QOL. This study aims to provide valuable insights to healthcare facilities offering PD, shedding light on its influence across various facets of a patient’s life, encompassing personal and occupational dimensions. Ultimately, our findings may contribute to cost savings, both for governments and individuals, by addressing the financial implications that may arise from lifestyle adjustments associated with the initiation of PD.

## Methods

### Study design

A cross-sectional study was conducted between October 2021 and January 2022 at An-Najah National University Hospital (NNUH) in Nablus, Palestine. The study included 74 patients who were receiving PD and were between 18 and 85 years old and had been receiving PD for at least 3 months. Patients who had received PD for less than 3 months and those under 18 years old were excluded from the study. The study protocol was approved by the Institution Review Board (IRB) at Al Najah National University and informed consent forms were signed by the participants.

### Variables and data collection tools

#### Dependent variables

The Malnutrition-Inflammation Score (MIS) rates inflammation and protein-energy wasting on a scale of 0 to 30. The results were calculated using an online calculator available at this website: http://www.touchcalc.com/calculators/mis. It comprises ten components divided into four sections: nutritional history, physical examination, BMI, and laboratory values. Each component has four levels of severity, ranging from 0 (normal) to 3 (severely abnormal). The following characteristics were considered when calculating the MIS score: change in weight, nutritional intake, gastrointestinal (GI) symptoms, functional capacity, co-morbidities, body composition, muscle wasting, BMI, serum albumin, and total iron-binding capacity. The total of all ten MIS components can vary from 0 (normal) to 30 (severely malnourished). A higher score indicates more severe levels of malnutrition and inflammation.

The QOL score is a tool for assessing an individual’s quality of life across five domains: physical, psychological, social, economic, and spiritual. It evaluates the ability to perform tasks like walking, self-care, work, studying, or chores, as well as the experience of pain, discomfort, depression, or anxiety. Each item is scored from 0 (indicating poor health) to 4 (indicating good health). The scores for each item are then summed, and the domain scores are transformed to a 0-100 scale.

#### Independent variables

Numerous characteristics were collected to achieve the study’s objectives, including age in years, gender (male or female), place of residency (camp, village, or city), occupation, income, marital status, kidney transplant history (yes or no), ability to self-administer medication (yes or no), smoking status (yes or no), hypertension (yes or no), presence of pitting edema (yes or no), living arrangements (alone or with family), duration of dialysis (in years), dialysis frequency (per day), BMI, and other relevant factors.

### Data analysis

R version 4.1.1 (https://www.r-project.org) was utilized for data analysis. Group comparisons between the Malnutrition-Inflammation Score (MIS) and both quantitative and qualitative variables were conducted using the ANOVA test, Mann–Whitney test, or Kruskal test, depending on the statistical distribution of the variables. Pearson correlation (r) was employed to examine the association between MIS and quantitative features. Univariate and multivariate linear regression analyses were conducted to assess the relationship between QOL and MIS scores. In the multivariate analysis, MIS was adjusted for variables such as Dialysis period (years), diabetes, hypertension, living arrangements, and the presence of pitting edema. A two-sided P value of < 0.05 was considered indicative of statistical significance. In the case of qualitative variables, some numerical features were also categorized, including age and BMI.

## Results

### Demographics and characteristics of the study participants

The study involved 74 participants, with a mean age of 50.5 ± 16.38 years. Approximately 36.49% were over 60 years old, and 55.41% were female. The majority (47.3%) resided in villages, followed by those in cities (44.59%), and the remainder in camps. A significant portion (77.03%) of the participants were unemployed, while a minority (24.32%) had completed undergraduate studies. Most participants (63.51%) had completed secondary education, with a small percentage having graduate education (8.11%) and the same percentage having no education beyond secondary school. Only 10.81% had received a kidney transplant, and 83.78% could manage their medication independently. Smoking was reported by 25.68% of participants. Over two-thirds (67.57%) were married, and more than half (51.35%) had been on dialysis for over a year. The majority (82.43%) underwent dialysis more than four times a day.

Among the participants, the majority had a healthy weight (37.84%), while 32.43% were overweight, 22.97% were obese, and 6.76% were underweight. Approximately 54.05% had an income of less than 2000 NIS, 43.24% had incomes ranging from 2000 to 5000 NIS, and only two patients had incomes higher than 5000 NIS. About 32.34% of patients had mild pitting edema, and the vast majority (93.24%) lived with their families. Additionally, 64.86% had hypertension, and 39.19% were diabetic (Table 1).

### MIS score of participants

The average MIS score for all participants was 7.5 ± 3.45. The results indicated a positive correlation between age and MIS (r = 0.2, *p* = 0.09), while Dialysis frequency/day (r = -0.03, *p* = 0.82) and BMI (r = -0.06, *p* = 0.58) showed negative correlations with MIS. Other variables demonstrated significant associations with MIS scores, including the ability to take medication independently (*p* = 0.04), mild pitting edema (*p* < 0.001), and diabetes (*p* < 0.001). Participants who couldn’t take their medication alone had a higher MIS score with a mean of 9.5 ± 3.32. Mild pitting edema was associated with higher MIS scores, with a mean of 9.46 ± 3.27, and diabetes was also associated with a higher MIS score, with a mean of 9.14 ± 3.52 (See Table 1, columns 3–7).

**Table 1:** Population characteristics for participants and group comparison by MIS and QOL scores

Variable	n(%)	MIS			QOL		
		r[*p*]	Mean ± SD	*p*	r[*p*]	Mean ± SD	*p*
**MIS**							
n (Missing)	74(0)	1[< 0.001]			-0.65[< 0.001]		
Mean ± SD	7.5 ± 3.45						
min, max	1,15						
**QOL**							
n (Missing)	74(0)	-0.65[< 0.001]			1[< 0.001]		
Mean ± SD	73.92 ± 27.06						
Median(Q1-Q3)	85(61.25-95)						
min, max	0,100						
**Age**							
n (Missing)	74(0)	0.2[0.09]			-0.33[< 0.001]		
Mean ± SD	50.5 ± 16.38						
min, max	18,85						
**Age**							
< 60	47(63.51%)		7.02 ± 3.51	0.09^b^		80 ± 22.53	0.01^a^
≥ 60	27(36.49%)		8.33 ± 3.25			63.33 ± 31.22	
**Gender**							
Female	41(55.41%)		7.54 ± 3.74	0.89^b^		77.44 ± 26.25	0.21^a^
Male	33(44.59%)		7.45 ± 3.12			69.55 ± 27.82	
**Residency**							
Camp	6(8.11%)		7.83 ± 3.92	0.39^c^		69.17 ± 33.83	0.81^c^
Village	35(47.3%)		8.06 ± 3.72			70.43 ± 30.06	
City	33(44.59%)		6.85 ± 3.05			78.48 ± 22.2	
**Occupation**							
Employed	17(22.97%)		6.18 ± 3.76	0.09^b^		89.12 ± 18.05	0.01^a^
Unemployed	57(77.03%)		7.89 ± 3.29			69.39 ± 27.76	
**Education**							
None	6(8.11%)		8.33 ± 3.78			45 ± 32.25	0.84^c^
Secondary	47(63.51%)		7.89 ± 3.28			72.98 ± 27.79	
Under graduate	18(24.32%)		6.39 ± 3.71			84.44 ± 14.84	
Graduate	3(4.05%)		6.33 ± 4.04	0.20^c^		83.33 ± 28.87	
**Income**							
< 2000	40(54.05%)		8.28 ± 3.34	0.28^c^		63.38 ± 29.69	0.19^c^
2000–5000	32(43.24%)		6.62 ± 3.48			85.94 ± 17.25	
5000–10,000	1(1.35%)		8 ± 0			85 ± 0	
> 10,000	1(1.35%)		4 ± 0			100 ± 0	
**Smoke**							
No	55(74.32%)		7.35 ± 2.94	0.55^b^		75.64 ± 24.96	0.36^a^
Yes	19(25.68%)		7.95 ± 4.71			68.95 ± 32.64	
Living arrangement							
Alone	5(6.76%)		6.2 ± 2.49	0.38^b^		65 ± 12.75	0.12^b^
With family	69(93.24%)		7.59 ± 3.51			74.57 ± 27.76	
**Marital status**							
Single	24(32.43%)		7.08 ± 3.63			73.33 ± 29.59	0.90^a^
Married	50(67.57%)		7.7 ± 3.38	0.48_b_		74.2 ± 26.08	
**Dialysis period (Year)**							
< 1	36(48.65%)		7.61 ± 3.6	0.66^b^		75.69 ± 28.31	0.59^a^
>=1	38(51.35%)		7.39 ± 3.36			72.24 ± 26.09	
**Dialysis frequency (day)**							
n (Missing)	74(0)	-0.03[0.82]			-0.05[0.67]		
Mean ± SD	3.91 ± 0.53						
min, max	3,6						
**Dialysis frequency (day)**							
< 4	13(17.57%)		9 ± 3.27	0.07^b^		68.08 ± 31.46	0.40^a^
>=4	61(82.43%)		7.18 ± 3.43			75.16 ± 26.16	
**Kidney transplant**							
No	66(89.19%)		7.45 ± 3.5	0.75^b^		73.41 ± 26.76	0.64^a^
Yes	8(10.81%)		7.88 ± 3.18			78.12 ± 31.05	
**Taking medication alone**							
No	12(16.22%)		9.5 ± 3.32	0.04^b^		40 ± 28.68	< 0.001^b^
Yes	62(83.78%)		7.11 ± 3.37			80.48 ± 21.4	
**Pitting edema**							
Mild	24(32.43%)		9.46 ± 3.27	< 0.001^b^		57.08 ± 32.37	< 0.001^a^
None	50(67.57%)		6.56 ± 3.16			82 ± 19.85	
**Hypertension**							
No	26(35.14%)		7.04 ± 3.22	0.45^b^		75.77 ± 26.18	0.67^a^
Yes	48(64.86%)		7.75 ± 3.58			72.92 ± 27.75	
**Diabetic**							
No	45(60.81%)		6.44 ± 3	< 0.001^b^		84 ± 15.02	< 0.001^a^
Yes	29(39.19%)		9.14 ± 3.52			58.28 ± 33.73	
**BMI**							
n (Missing)	74(0)	-0.06[0.58]			-0.24[0.04]		
Mean ± SD	26.22 ± 6.2						
min, max	14.68,44.95						
**BMI**							
Healthy Weight	28(37.84%)		7.64 ± 3.91	0.90^c^		74.82 ± 26.99	0.79^c^
Obese	17(22.97%)		8.65 ± 3.2			57.35 ± 29.27	
Over Weight	24(32.43%)		6 ± 2.8			82.08 ± 23.68	
Under Weight	5(6.76%)		10 ± 1.22			86 ± 6.52	

^a^ ANOVA test

^b^ Mann?Whitney U test

^c^ Kruskal?Wallis test

### MIS score relationship with the quality of life

The average QOL score was 73.96 ± 27.06 and had a significant negative association with the MIS score (r = -0.65, *p* < 0.001). This inverse relationship was particularly clear among participants with ages < 60 (MIS: 7.02 ± 3.51 vs. QOL: 80 ± 22.53), city residency (MIS: 6.85 ± 3.05 vs. QOL: 78.48 ± 22.2), employed subjects (MIS: 6.18 ± 3.76 vs. QOL: 89.12 ± 18.05), graduate educational group (MIS: 6.33 ± 4.04 vs. QOL: 83.33 ± 28.87), participants with an income of more than 10,000 NIS (MIS: 4 ± N/A vs. QOL: 100 ± N/A), non-smokers (MIS: 7.35 ± 2.94 vs. QOL: 75.64 ± 24.96), participants undergoing dialysis more than 4 times a day (MIS: 7.18 ± 3.43 vs. QOL: 75.16 ± 26.16), participants who underwent a kidney transplant (MIS: 7.88 ± 3.18 vs. QOL: 78.12 ± 31.05), participants without pitting edema (MIS: 6.56 ± 3.16 vs. QOL: 82 ± 19.85), non-hypertensive participants (MIS: 7.04 ± 3.22 vs. QOL: 75.77 ± 26.18), and non-diabetic participants (MIS: 6.44 ± 3 vs. QOL: 84 ± 15.02).

On the other hand, there was a direct relationship between MIS and QOL, indicating that lower MIS scores were associated with lower QOL scores. This was particularly evident in males (MIS: 7.45 ± 3.12 vs. QOL: 69.55 ± 27.82), those living alone (MIS: 6.2 ± 2.49 vs. QOL: 65 ± 12.75), those who were single (MIS: 7.08 ± 3.63 vs. QOL: 73.33 ± 29.59), those on dialysis for more than one year (MIS: 7.39 ± 3.36 vs. QOL: 72.24 ± 26.09), and those unable to take their medications independently (MIS: 9.5 ± 3.32 vs. QOL: 40 ± 28.68).

In the multivariate model, the MIS values were adjusted for other factors such as the duration of dialysis, the presence of diabetes and hypertension, living arrangements, and pitting edema. The results showed that MIS, diabetic subjects, and mild pitting edema were independently associated with a lower QOL score. Based on the standardized coefficient, diabetes had the strongest influence, followed by pitting edema. As clearly shown in Table 2, QOL had a significant negative association with MIS (B = -3.91, *P* < 0.001), diabetic subjects (B = -13.91, *P* = 0.01), and mild pitting edema (B = -11.09, *P* = 0.04), whereas it had a significant positive association with living alone (B = 19.33, *P* = 0.03).

**Table 2:** Univariate and multivariate linear regression for the association between QOL and MIS

Variables	Crude Β (95% CI)	*P*	Adjusted Β (95% CI)	*P*
MIS	-5.09 (-6.49, -3.7)	< 0.001	-3.91 (-5.42, -2.41)	< 0.001
Dialysis period /year	-3.46 (-16.07, 9.15)	0.59	-2.57 (-11.48, 6.35)	0.57
Diabetic	-25.72 (-37.16, -14.29)	< 0.001	-13.91 (-23.81, -4)	0.01
Hypertension	-2.85 (-16.06, 10.36)	0.67	1.31 (-8.1, 10.71)	0.78
Living arrangement (Alone)	9.57 (-15.49, 34.62)	0.45	-19.33 (1.45, 37.22)	0.03
Pitting edema (Mild)	-24.92 (-37.07, -12.76)	< 0.001	-11.09 (-21.45, -0.72)	0.04

## Discussion

This study marks the first-ever report on the quality of life among PD patients in Palestine. The findings show that PD patients living in the West Bank region have a relatively high average QOL score, at 73.92 ± 27.06. More than 50% of participants scored 85 or higher, indicating a pretty good QOL. Age and occupation turned out to be important factors related to QOL, each with a P-value of 0.01. The study also found that factors like a patient’s ability to self-administer medication, the presence of pitting edema, and diabetes mellitus were significantly linked to both MIS and QOL. But it’s worth noting that the study has a limitation – the relatively small sample size. Also, some patients might be a bit hesitant to choose PD for dialysis due to concerns about peritonitis. Since this study only looks at data collected at one point in time, it can’t definitively establish cause-and-effect relationships.

Previous research has shown strong links between QOL, MIS, and various related factors, highlighting how malnutrition can really impact a patient’s QOL [20–22]. In one study by Sohrabi et al., they looked at how malnutrition and inflammation affect the physical and mental aspects of health-related quality of life in HD patients. Two other studies had different goals: one checked the nutritional status of HD patients, and the other investigated how socio-demographic factors influence the nutritional status of Palestinian diabetic patients on HD therapy. The findings make it clear that there’s a significant negative link between MIS and QOL scores (*p* < 0.001), meaning that patients with poor nutritional status have lower QOL scores. This lines up with a previous study in Palestine, showing that malnutrition is tied to lower QOL scores in diabetic patients on HD [22]. This aligns with a prior study that demonstrated lower QOL scores among severely malnourished cancer patients when compared to those with milder malnutrition, emphasizing the significance of addressing malnutrition in healthcare [23].

On the other hand, our study showed a negative association between age and QOL, indicating that older age is associated with lower quality of life. Furthermore, around half of the participants (51.6%) were over 60 years old and were identified as being at risk for malnutrition. This finding aligns with a study conducted in Nepal involving 328 participants, which also identified a negative correlation between age and QOL [24]. Additionally, we observed a positive correlation between occupational and educational status and QOL, which mirrors the results of the Nepalese study, where individuals with higher occupational status and advanced educational degrees exhibited a notably higher QOL score [24].

The current study found a negative association between diabetes and quality of life, which is consistent with preceding studies [22, 25, 26]. A Spanish study reported that 58.1% of diabetic patients had a high risk of malnutrition and diabetes was associated with a lower quality of life [25]. The findings of this study are consistent with a study conducted at Birmingham Heartlands Hospital in the UK, which found that diabetic patients had lower scores on the Mini Nutritional Assessment compared to the control group [26]. This highlights the importance of proactive diabetes management for improved QOL of dialysis patients.

Likewise, the findings in this study point to a clear negative link between low QOL scores, older patients, and women. These results align with a Tanzanian study that found a significant drop in QOL as people get older, with women reporting lower QOL scores than men [27]. In addition, a study conducted in Nigeria suggested that social support has a greater impact on the QOL of older adults than health-related factors [28]. These results provide valuable insights into improving the well-being of specific patient demographics.

In light of these results, health practitioners should pay special attention to the nutritional status of patients, particularly those with diabetes or who are older in age. Assessing and addressing malnutrition, as well as providing support for diabetes management, can potentially improve the quality of life for these patients. Additionally, health professionals should consider patients’ occupational and educational backgrounds when tailoring their care plans, as these factors appear to play a role in determining quality of life. These insights can guide healthcare providers in offering more personalized and effective care to their patients.

## Conclusions

Based on this study, the quality of life (QOL) among peritoneal dialysis (PD) patients in Palestine is of particular significance. The research shows a relatively high average QOL score among PD patients in the West Bank, with over 50% experiencing favorable QOL. Age, occupation, medication dependency, and pre-existing conditions such as pitting edema and diabetes mellitus significantly impact QOL. Living with family positively correlates with QOL compared to living alone. These findings guide healthcare practitioners in enhancing PD patient care, emphasizing the importance of early detection of malnutrition, tailored approaches to nutritional support, and diabetes management for better QOL.

### Limitations

The study’s limitations include the absence of a control population not undergoing PD for result comparison, and the relatively small sample size, necessitating caution when interpreting the findings. Further research with a larger sample size and additional variables is recommended. Additionally, some patients may show hesitancy in selecting PD due to peritonitis concerns, and the study’s cross-sectional design precludes definitive cause-and-effect relationship establishment.

## Data Availability

The datasets used and/or analyzed during the current study are available from the corresponding author upon reasonable request.

## Abbreviations

CAPD: Continuous ambulatory peritoneal dialysis
CKD: chronic kidney disease
DM2: diabetes mellitus type 2
ESRD: End-stage renal disease
GFR: glomerular filtration rate
GI: Gastrointestinal
HD: hemodialysis
MIS: malnutrition-inflammation scale
PD: Peritoneal dialysis
QOL: quality of life
TIBC: total iron-binding capacity
